# Effects of chitosan combined with ε‐polylysine coating on flavor and texture quality of Chinese shrimp during refrigerated storage

**DOI:** 10.1002/fsn3.1432

**Published:** 2020-02-12

**Authors:** Zhen Zhang, Yunpo Huang, Xuesong Guo, Xin Meng, Handong Wu, Fang Guo, Shu‐ai Zhang, Dandan Li

**Affiliations:** ^1^ College of Food Science and Engineering Jinzhou Medical University Jinzhou China; ^2^ College of Public Basic Sciences Jinzhou Medical University Jinzhou China

**Keywords:** Chinese shrimp, chitosan, coating, flavor, storage, texture

## Abstract

We investigated the effects of chitosan (CH) combined with ε‐polylysine (ε‐PL) on the flavor and texture quality of Chinese shrimp refrigerated for 12 days. Shrimp samples were subjected to three preservation treatments (ε‐PL, CH, and CH + ε‐PL) and a control treatment. Sensory characteristics, total volatile basic nitrogen (TVB‐N), adenosine triphosphate (ATP)‐related compounds, K‐values, volatile components, and texture were regularly assessed. The results showed that the sensory characteristics were effectively maintained, the increases in TVB‐N, hypoxanthine, and K‐value were delayed, and the putrid compounds were reduced by coating, especially with the chitosan combined with ε‐polylysine. Treatment with chitosan combined with ε‐polylysine was also shown to be a more effective preservation method for maintaining the texture quality of Chinese shrimp compared to treatment with ε‐PL or CH alone. Therefore, this technique was demonstrated to be a promising method for maintaining the flavor and texture quality of Chinese shrimp during refrigerated storage.

## Graphical abstract

The relative content of off‐flavor compounds were reduced by chitosan and ε‐polylysine coating. The increases in TVB‐N, hypoxanthine, and K‐value were delayed by treatment with chitosan combined with ε‐polylysine. Treatment with chitosan coating combined with ε‐polylysine was a promising method for maintaining the flavor and texture quality of Chinese shrimp during refrigerated storage.

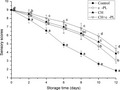

## 1 INTRODUCTION

As the most widely distributed shrimp in China, Chinese shrimp (*Fenneropenaeus chinensis*) is an ideal food product with important implications for human health because it has a high content of polyunsaturated fatty acids, and various vitamins and essential trace elements (Lu, 2009) and also has a high protein content, a low fat content, and tender and delicious meat (Dayal et al., 2013). Therefore, Chinese shrimp has important economic value and has become one of China's important export aquatic products (Anonymous, 2018). However, Chinese shrimps are easily perishable owing to their high protein and water contents, autolysis, and microbial metabolism, and the changes in Chinese shrimps caused by the degradation results in the accumulation of putrid compounds and a deterioration in texture quality (Bahmani et al., 2011; Li, Yang, & Li, 2017; Nirmal & Benjakul, 2009). The original flavor of Chinese shrimp also lost along with the production of off‐odor compounds. Consequently, the market value is significantly reduced. Therefore, preventing shrimp spoilage and maintaining the flavor and texture quality of shrimp is of great importance. Several researches have been carried out on the improvement of shrimp quality, and coating, as one of the most effective ones, are attracting increasing attention owing to their effectiveness, low cost and safety and have proven to be effective for maintaining the quality of shrimp. Studies have increasingly been performed on extending the shelf ‐lives of shrimp, fish, and other seafood using natural extracts with antimicrobial and antioxidant properties coatings rather than synthetic additives (Rodriguez‐Turienzo et al., 2011; Yu, Regenstein, et al., 2018; Yu, Xu, et al., 2018).

Chitosan (CH), a widely present polysaccharide, has been extensively used in seafood preservation. It can be absorbed by the body and is the only high‐molecular‐weight alkaline polysaccharide that is widely abundant in nature (Aider, 2010; Duan, Cherian, & Zhao, 2010). Because of its excellent film‐forming and antibacterial characteristics (Günlü & Koyun, 2013), capability of preventing microbial degradation, and biocompatibility, chitosan coating has been reported the potential as a technique to maintain the seafood quality by inhibiting the growth of microorganism, decomposition of protein, and oxidation of lipid during storage (Dehghani, Hosseini, & Regenstein, 2018; Fan et al., 2009; Ojagh, Rezaei, Razavi, & Hosseini, 2010). Ε‐polylysine (ε‐PL), as a natural preservative, has also been used in the preservation of seafood (Li et al., 2014; Najjar, Kashtanov, & Chikindas, 2007) because of its antibacterial properties (Geornaras, Belk, Smith, & Sofos, 2007; Zhou et al., 2011), nontoxicity, and biocompatibility (Hiraki, 2000). In addition, ε‐polylysine is a nutrient‐type bacteriostatic agent that can be decomposed into lysine, one of the eight essential amino acids (Yoshida & Nagasawa, 2003). In Japan, ε‐polylysine has been approved as a food preservative.

At present, Chitosan or ε‐polylysine has been studied as an effective measure to ensure the safety and extended the shelf‐life of seafood by evaluating microbial change, K‐value, thiobarbituric acid, and so on. In addition, Chitosan combined with ε‐polylysine has been used in preservation of white blushing carrots; however, only a few studies have investigated the flavor and texture quality of seafood products using a coating of chitosan combined with ε‐polylysine. Therefore, in this study, the effectiveness of a coating of chitosan and ε‐polylysine on the flavor and texture quality of Chinese shrimp was explored by analyzing the sensory characteristics, total volatile basic nitrogen (TVB‐N), adenosine triphosphate (ATP)‐related compounds, volatile components, and texture.

## 2 MATERIALS AND METHODS

### 2.1 Samples and chemicals

Chinese shrimp (*Fenneropenaeus chinensis*), of a size equivalent to 50–55 shrimps/kg, were obtained from the Jinzhou aquatic market, Liaoning, China, and transferred to the Food Processing Laboratory of Jinzhou Medical University within 30 min, so that they remained alive. After killing, washing, and draining them, the shrimp samples were randomly divided into four treatment groups. The chemicals used included chitosan (85% deacetylation degree, food grade; Zhejiang Aoxing Biotechnology Co., Ltd) and ε‐polylysine (Shandong Xin Ding Biotechnology Co., Ltd).

To prepare the coating solutions, chitosan (20.0 g) was poured into a flask containing 1% (v/v) acetic acid (700 ml). It was stirred until it dissolved completely; then, the volume was filled to 1,000 ml. The ε‐polylysine solution was obtained by dissolving ε‐polylysine (10.0 g) in 1,000 ml of distilled water. To obtain the CH + ε‐PL coating solution containing 2.0% chitosan and 1.0% ε‐polylysine, 10.0 g of ε‐polylysine was mixed with the prepared CH coating solution.

The Chinese shrimp samples were dipped in solutions containing 2.0% chitosan (CH group), 1.0% of ε‐polylysine (ε‐PL group), 2.0% chitosan and 1.0% ε‐polylysine (CH + ε‐PL group), and distilled water (control group) for 1 hr. After they were drained, the samples were packed in airtight polyethylene pouches and stored at 4°C. The sensory characteristics, TVB‐N, ATP‐related compounds, and texture were analyzed every 2 days, and the volatile components were evaluated every 6 days.

### 2.2 Sensory evaluation

The sensory evaluation was performed by the Meilgaard, Civille, and Carr (1990) method, and sensory scores were calculated by a well‐trained panel of seven evaluators. Each evaluator rated the characteristics from 1 (lowest quality) to 9 (most ideal) with regard to color, texture, odor, and overall acceptability of the samples. The shrimp was considered acceptable until the sensory score dropped to 5.0.

### 2.3 Total volatile basic nitrogen

The TVB‐N was measured according to the Chinese National Standard (GB 5009.228‐2016). Briefly, 10 g of crushed shrimp was mixed with 100 ml of distilled water in a conical flask, followed by homogenization and filtering. Next, 5 ml of the filtrate and 5 ml of an MgO suspension were injected into a reaction chamber. A boric acid solution (10 ml) and a mixture of methyl red and methylene blue (v:v = 2:1) were added, followed by distillation. Subsequently, the boric acid solution was titrated with a 0.1 M HCl solution. The TVB‐N value, which was expressed in mg nitrogen (mg/100 g) 100 g‐1 shrimp sample, was determined according to the consumption of the HCl solution.

### 2.4 ATP‐related compounds and K‐value

In accordance with the China Fisheries Industry Standard (SC/T 3048‐2014), ATP‐related compounds were extracted from the Chinese shrimp as follows. The homogenized sample (2 g) and 10% perchloric acid solution (20 ml) was mixed, vortexed for 1 min, and centrifuged at 1940*g* for 10 min at 4°C. After the supernatant was collected, the analyte was extracted from the precipitate twice under the aforementioned process parameters. The pH of the combined supernatant was adjusted within a range of 6.0–6.4 using 1.0 M NaOH. The pH‐adjusted solution was transferred to a precooled volumetric flask (volume of 50 ml), centrifuged at 1940*g* for 10 min at 4°C, and filtered using a 0.22 μm microporous membrane filter.

The following high‐performance liquid chromatography conditions were used for the ATP correlation analyses: C_18_ column (250 × 4.6 mm, 5 μm); mixed with a solution containing 0.02 M potassium dihydrogen phosphate and 0.02 M dipotassium phosphate 1:1 liquid as the mobile phase; the flow rate of the sample (10 µl) was maintained at 1 ml/min, and the peak was detected at 254 nm.

The K‐value was defined as the ratio of the total amount of inosine and hypoxanthine degraded by inosine triphosphate to the total amount of adenosine triphosphate‐related compounds as follows:

$$K\;-\;\text{value}\;\%=\frac{\text{MHxR}+\;\text{MHx}}{\text{MATP}+\;\text{MADP}+\;\text{MAMP}+\;\text{MIMP}+\text{MHxR}+\;\text{MHx}}\times 100$$

HxR: hypoxanthine adenosine, Hx: hypoxanthine, ATP: adenosine triphosphate, ADP: adenosine 5′‐diphosphate, AMP: adenosine 5′‐monophosphate, IMP: inosine 5′‐monophosphate.

### 2.5 Headspace SPME‐GC/MS analysis

The head, tail, and shell of the shrimp were removed and then crushed. Three grams of the sample was weighed and placed in the headspace sampler. The samples were held for 5 min and extraction was conducted for 40 min at 40°C. Analysis and identification were performed by GC‐MS after desorption for 5 min. The chromatographic conditions of the HP‐5 MS (30 m × 250 μm × 0.25 μm) column were used. The temperature was initially maintained at 40°C for 5 min and then increased to 220°C at 5°C/min. The injection port temperature was 250°C; the flow rate of the carrier gas (Helium) was 0.9 ml/min. The EI ionization mode was used for mass spectrometry. The electron energy was 70 eV, and the detector voltage was 350 V; the scanning ranged from 35 to 450 m/z. The temperatures of the ion source and interface were set at 220°C and 250°C, respectively.

### 2.6 Texture analysis

Texture analysis was conducted by evaluating the shear force of Chinese shrimp using a TA.XT Texture Analyzer (Stable Micro Systems Ltd). Ten samples from five shrimps were used for analysis. The parameters were set as follows: trigger force, 5 g; test speed, 1 mm/s; sample deformation degree, 50%.

### 2.7 Statistical analysis

Measurements, except those of sensory characteristics and TPA, were carried out with three replicates. Data, except for the VOC, were expressed as the mean ± standard deviation. Analyses were performed using SPSS software (version 19.0; SPSS). A value of *p* < .05 was considered statistically significant.

## 3 RESULTS AND DISCUSSION

### 3.1 Sensory analyses

**Figure 1:**
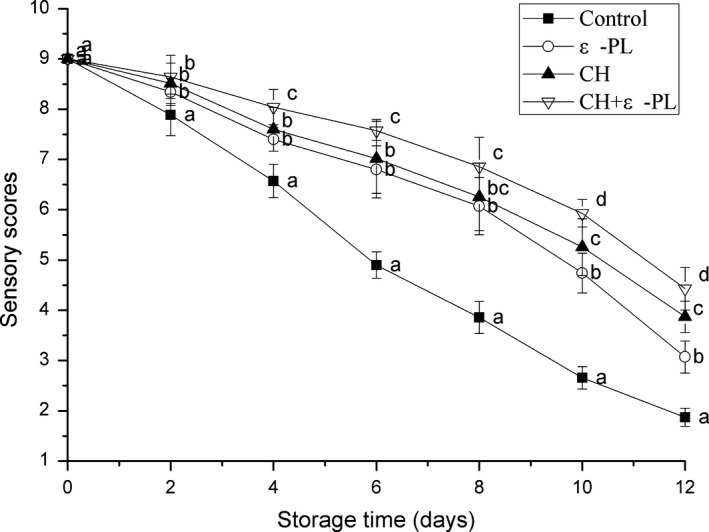
Changes in sensory scores of Chinese shrimp during refrigerated storage. Bars indicate means ± *SD* of seven replicates. Different letters for the data points at each sampling point indicate significant differences (*p* < .05)

The quality of the shrimp can be indicated by the sensory profile during storage. According to the Meilgaard, Civille, and Carr, shrimp is considered acceptable if the sensory score is ≥5.0. In the present study, as shown in Figure 1, the sensory scores of the Chinese shrimp in the different treatment groups decreased with prolonged storage. The samples in the control group exhibited the most rapid reduction in the sensory scores. In the present study, the control group was considered inedible on the 6th day, when the sensory score decreased to 4.90 ± 0.26. Li et al. (2017) also reported a similar result; the sensory quality of Pacific white shrimp was unacceptable on the sixth day. In contrast, the sensory scores of the ε‐PL and CH groups were significantly (*p* < .05) higher and unacceptable (4.74 ± 0.40, 3.87 ± 0.31) on day 10 and day 12, respectively. These above results are attributed to the protective coating comprising chitosan or chitosan and ε‐polylysine on the surface of the shrimp, which inhibited microbial proliferation and protein degradation and minimized the production of volatile substances. Among all the treatment groups, the samples in the CH + ε‐PL group exhibited the highest sensory scores during the storage; their scores reached an unacceptable value (4.43 ± 0.42) on day 12. The improved sensory effect was attributed to the coating performance and bacteriostasis due to the addition of ε‐polylysine to the chitosan. Yu, Xu, et al. (2018) reported that the sensory quality of grass carp fillets could be better maintained by the chitosan composite with essential oil than chitosan alone.

### 3.2 Total volatile basic nitrogen

**Figure 2:**
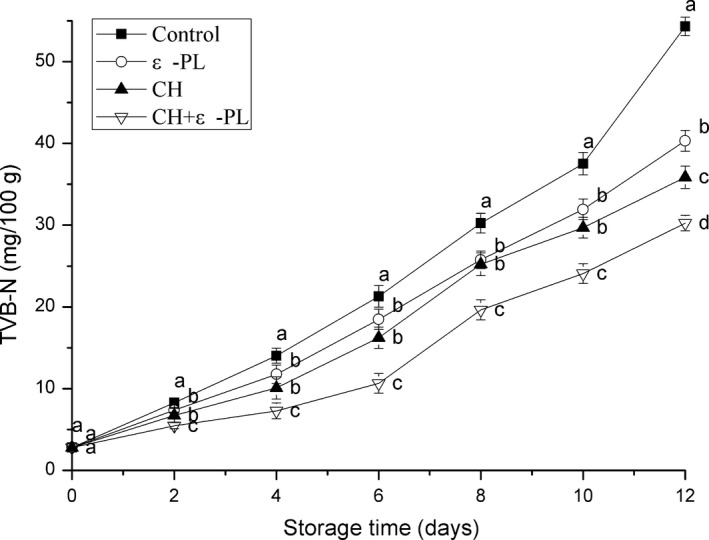
Changes in the total volatile basic nitrogen values of Chinese shrimp during refrigerated storage. Bars indicate means ± *SD* of three replicates. Different letters for the data points at each sampling point indicate significant differences (*p* < .05)

Total volatile basic nitrogen, referring to a class of volatile basic nitrogenous compounds including dimethylamine, trimethylamine, and ammonia, is an important indicator for evaluating the freshness of aquatic products (Hui, Liu, Feng, Li, & Gao, 2016). A high TVB‐N value reflects a decrease in the flavor quality of shrimp. According to the hygienic standards for marine products, the acceptable TVB‐N values for marine fish and shrimp are ≤30 mg/100 g (GB 2733‐2015). As shown in Figure 2, the TVB‐N values continuously increased from 2.81 ± 0.17 mg/100 g for all the samples with prolonged storage; however, it was significantly faster (*p* < .05) for the control group than for the treatment groups. For the control group, it reached 30.25 ± 1.20 mg/100 g on the 8th; this was significantly higher (*p* < .05) than the values for the treated groups (25.76 ± 1.07, 25.22 ± 1.40, 19.63 ± 1.23 mg/100 g for ε‐PL, CH, CH + ε‐PL, respectively). The TVB‐N value of the shrimp samples in the CH group eventually reached 36.91 ± 1.46 on day 12, which was slightly lower than that of the ε‐PL group (41.66 ± 1.02 mg/100 g). The lowest TVB‐N values were observed for CH + ε‐PL group: 25.11 ± 1.20 mg/100 g on the 10th day and 31.49 ± 1.16 mg/100 g on the 12th day. For the control group, the TVB‐N values reached 54.32 ± 1.13 mg/100 g on the 12th day. The TVB‐N in postmortem shrimp is primarily produced via the microbial degradation of proteins and other nitrogen‐containing compounds (Li et al., 2012; Song, Liu, Shen, You, & Luo, 2011). In the present study, the CH + ε‐PL group had the lowest TVB‐N values because that chitosan and ε‐polylysine significantly inhibited the bacterial growth and reduced the oxidative deamination of nitrogen‐containing compounds by bacteria. The foregoing results are consistent with previous studies, wherein the TVB‐N values of silver carp fillets treated with chitosan were found to be lower (Ramezani, Zarei, & Raminnejad, 2015).

### 3.3 ATP‐related compounds and K‐value

**Figure 3:**
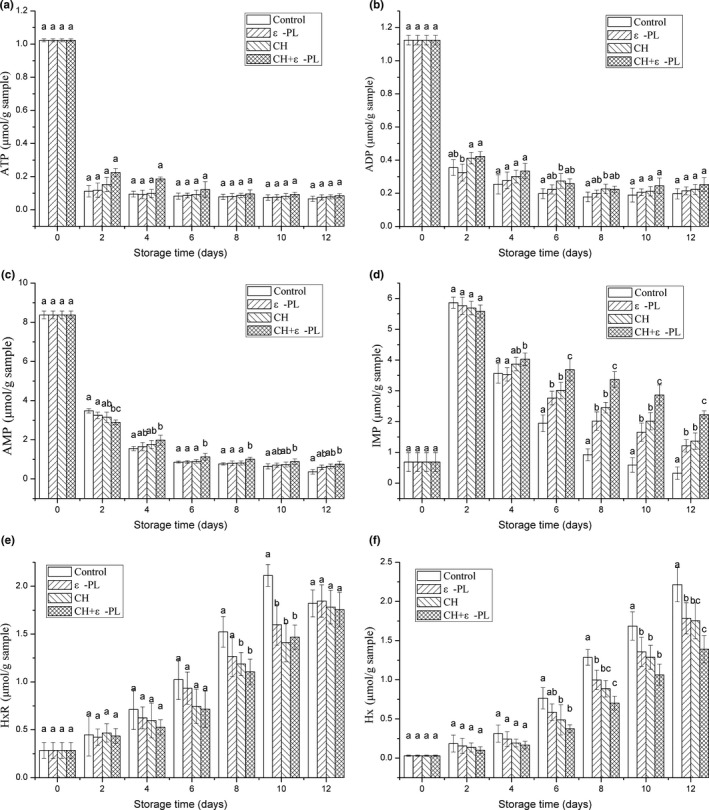
(a–f) Changes in the ATP‐related compounds of Chinese shrimp samples during refrigerated storage. Bars indicate means ± *SD* of three replicates. Different letters for the data points at each sampling point indicate significant differences (*p* < .05)

As shown in Figure 3a–d, in general, the contents of ATP, ADP, and AMP in shrimp of the four groups showed similar trends that decreased rapidly in the first 4 days and then more gradually. The ATP content in the samples of control, ε‐PL, CH, and CH + ε‐PL decreased by 93.53%, 92.65%, 92.35%, and 91.67% at the end of storage, respectively. Correspondingly, the ADP content decreased by 82.47%, 80.87%, 80.16%, and 77.58%. The rapid degradation of ATP in the shrimp after death is related to the high activity of ATP‐degrading enzyme in vivo. Among all the ATP‐related compounds, AMP was the most important nucleic acid substance in fresh Chinese shrimp, with an initial content of 8.375 ± 0.195 μmol/g, which was significantly higher (*p* < .05) than that of the other nucleic acid metabolites. The results were similar to the findings of Mendes, Quinta, and Nunes (2001) for Norway lobster and red shrimp with an initial AMP value of 9.3–11.8 μmol/g. The high initial AMP value in fresh shrimp was mainly because of the low activity of the AMP‐degrading enzyme in the initial stage after the shrimp died (Yokoyama, Sakaguchi, Kawai, & Kanamori, 1992). After 12 days, the AMP value in CH + ε‐PL dropped to 0.754 ± 0.140 μmol/g, which was significantly higher (*p* < .05) than that of the control. As an important umami substance of shrimp, IMP increased rapidly in the early stage of storage with the decomposition of ATP and then decreased gradually after reaching the peak. The rate of decline was faster in the control group than in the treatment group, and the IMP content reduced to 0.324 ± 0.203 μmol/g on day 12. These were 73.27%, 76.26%, and 85.44% lower than those in ε‐PL, CH, CH + ε‐PL treatment, respectively. Compared with ε‐PL or CH, the IMP content was significantly higher (*p* < .05) in CH + ε‐PL. The changes in the IMP content indicated that the activities of autolytic and microbial enzymes were associated with AMP degradation and that further decomposition of IMP was effectively suppressed by the treatment with chitosan, ε‐polylysine, or chitosan combined with ε‐polylysine.

As off‐odor substances, the accumulation of HxR and Hx affected the flavor quality of shrimp. As shown in Figure 3e–f, the HxR contents of the control increased until its peak (2.112 ± 0.115 μmol/g), and then dropped to 1.821 ± 0.140 μmol/g, while a continuous increase was observed with the treatments. The reason for the decrease in the control might be the decomposition of HxR by the bacteria (Yu, Xu, et al., 2018). The peak values for the ε‐PL, CH, and CH + ε‐PL groups were 12.6%, 20.7%, and 25.2% lower than the control, respectively. Conversely, the initial Hx content (0.030 ± 0.008 μmol/g) was significantly lower (*p* < .05) than that of HxR (0.285 ± 0.083 μmol/g), increased slowly for the first 4 days, and then rapidly. There were no significant differences (*p* ≥ .05) among the four groups until day 6. The final Hx concentration in the control reached 2.214 ± 2.218 μmol/g, while it was significantly lower (*p* < .05) in the treated groups. These results suggested that the coating treatment could effectively maintain the flavor quality of Chinese shrimp, and the effect was enhanced by the combination of chitosan with ε‐polylysine.

**Figure 4:**
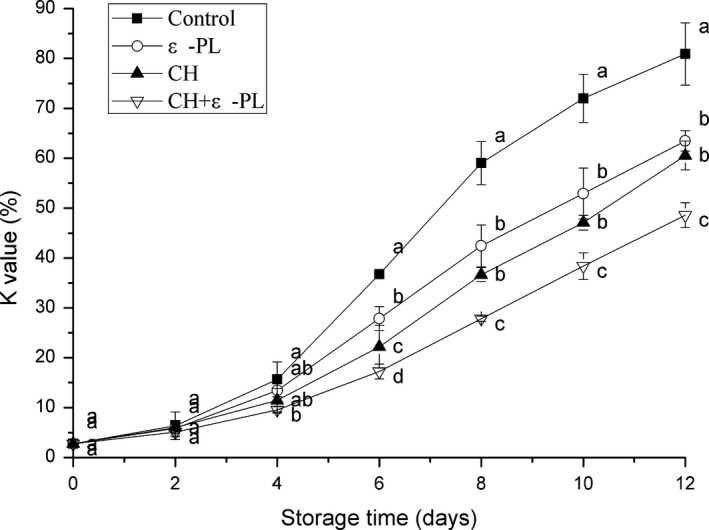
Changes in the K‐values of Chinese shrimp samples during refrigerated storage. Bars indicate means ± *SD* of three replicates. Different letters for the data points at each sampling point indicate significant differences (*p* < .05)

The K‐value is the ratio of the total amount of inosine and hypoxanthine degraded by inosine triphosphate to the sum of ATP‐related compounds. This index and content of ATP‐related compounds have been frequently used to evaluate the freshness of seafood (Ocaño‐Higuera et al., 2011; Pardio, Waliszewski, & Zuñiga, 2011). In previous studies, seafood with K‐values of ＞60%, 20%‐50%, and ≤20% were considered as not fresh, moderately fresh, and very fresh, respectively (Ehira, 1976; Saito, Arai, & Matsuyoshi, 1959). As shown in Figure 4, the K‐value was 2.73%±0.74% on day 0, which was slightly higher than the value of 1.80% reported by Ando, Nakamura, Harada, and Yamane (2004), and it reached 80.91%±6.25% on the 12th day. Various factors (processing temperature, stress during capture, the species, and muscle type) lead to the differences in the K‐values of aquatic products (Alasalvar et al., 2001; Sallam, 2007). A gradual increase in the K‐value was observed for both the treated and control groups during the 12‐day storage period; however, the K‐values of the samples in the treated groups were significantly lower (*p* < .05) than those of the control samples on day 12. The K‐value in the control group was 72.01% ± 4.85% on day 10, which cannot be considered fresh; the K‐values in the ε‐PL and CH groups were 63.47% ± 2.04% and 60.54% ± 2.89%, respectively, on day 12; while the samples in the CH + ε‐PL group were moderately fresh at the end of the storage period, indicating that the ATP degradation was effectively inhibited and the shelf‐life of the shrimp samples was extended by chitosan and ε‐polylysine.

### 3.4 Headspace SPME‐GC/MS analysis

In this study, hydrocarbons were not considered because of the high flavor threshold and less impact on the overall flavor of aquatic products. The peak area changes of 44 main VOCs in Chinese shrimp are listed in Table 1. These detected compounds include aldehydes (11), alcohols (11), ketones (9), esters (4), and N/S‐Containing (9). On day 0, 15 compounds were detected, and the characteristic flavor substances of fresh Chinese shrimp detected more than others were aldehydes and alcohols. Among them, 2‐ethyl‐1‐hexanol, 1‐octen‐3‐ol, hexanal, and nonanal had larger peak areas. During the storage period, the peak areas of many compounds increased, decreased, or disappeared, and new compounds were detected. At the end of storage, there were 40, 35, 24, and 18 compounds detected in control, ε‐PL, CH, and CH + ε‐PL, respectively.

**Table 1:** Main volatile compounds and their relative contents (chromatographic peak area × 10^−7^) in Chinese shrimps at storage days 0, 6, and 12

Compound	d0	d6				d12			
		Control	ε‐PL	CH	CH + ε‐PL	Control	ε‐PL	CH	CH + ε‐PL
Aldehydes (11)									
(E,E)‐2,4‐Heptadienal	–	0.06	0.07	0.02	–	7.55	6.86	1.23	–
3‐Methylbutyraldehyde	––	0.28	0.12	0.05	–	13.56	7.86	3.17	1.01
2‐Methylbutyraldehyde	–	0.13	0.09	0.06	–	10.04	5.34	2.13	0.66
Pentanal	0.06	0.21	–	–	–	0.36	0.11	–	–
Hexanal	1.86	4.29	1.59	1.33	1.03	15.05	6.12	2.23	1.01
Octanal	0.05	0.10	–	–	–	0.43	–	–	–
Nonanal	1.39	1.46	0.86	1.55	1.15	0.93	0.52	0.63	0.62
Hexadecanal	0.05	0.68	0.08	–	–	1.12	0.02	–	–
Decanal	0.07	0.44	–	–	–	1.54	0.05	–	–
2‐Methyl‐3‐phenyl‐Propanal	–	–	–	–	–	0.11	0.03	–	–
Octadecanal	–	0.44	–	–	–	–	0.02	–	–
Alcohols (11)									
Octanol	0.78	1.23	1.02	0.58	0.24	0.61	0.82	0.25	0.09
Phenethyl alcohol	–	2.21	1.11	1.21	–	20.35	10.22	6.83	1.96
1‐Butanol	–	0.05	0.02	–	–	0.08	0.03	–	–
2,2‐Dimethyl‐1‐butanol	–	0.06	0.07	0.04	–	3.65	1.38	0.33	–
3‐Methyl‐1‐butanol	–	0.58	0.65	0.12	–	5.65	3.38	1.33	0.89
2‐Ethyl‐1‐Hexanol	1.75	11.32	8.35	4.25	0.23	24.65	15.56	8.97	1.08
1‐Hexanol	0.89	1.58	1.29	0.36	0.17	1.14	0.93	0.58	0.25
1‐Octen‐3‐ol	1.65	4.48	3.64	1.12	0.86	2.26	1.62	0.85	0.14
1‐Pentanol	0.02	0.11	–	0.02	–	0.17	0.05	0.03	–
1‐Penten‐3‐ol	0.62	0.52	0.54	0.06	0.11	0.56	0.32	0.13	0.19
2‐Hexanethiol	–	–	–	–	–	0.02	–	–	–
Ketones (9)									
2‐Methyl‐1‐hepten‐6‐one	–	0.06	0.03	–	–	0.05	–	–	–
2‐Heptanone	–	0.99	0.38	0.12	–	1.65	0.87	0.45	–
2‐Nonanone	1.71	1.93	0.88	1.01	0.24	3.15	1.87	1.28	0.46
Cyclohexanone	–	0.05	–	–	–	–	0.03	–	–
2‐Octanone	0.03	0.02	–	–	–	–	–	–	–
3,5‐Hexadiyn‐2‐one	–	0.02	–	–	–	0.05	–	–	–
(E,E)‐3,5‐Octadien‐2‐one	–	0.03	–	–	–	0.08	–	–	–
2,4‐Dimethyl‐3‐hexanone	–	–	–	–	–	–	0.02	–	–
6‐Methyl‐5‐hepten‐2‐one	–	0.04	0.02	0.02	0.03	0.05	0.03	0.04	0.06
Esters (4)									
Ethyl acetate	–	0.11	–	–	–	0.23	0.05	–	–
11‐Methyl‐dodecanoate	–	0.16	0.11	–	–	0.21	0.15	0.03	–
1‐Octen‐3‐yl‐acetate	–	0.03	–	–	–	0.07	–	–	–
2,4‐Di‐tert‐butylphenyl benzoate	–	–	0.02	–	–	0.07	–	–	–
N/S‐Containings (9)									
Trimethyl amine	–	0.65	0.38	0.11	–	3.92	1.28	0.89	0.22
3‐Methylpyridazine	–	0.08	0.04	–	–	0.22	0.09	–	–
Pyrazine	–	6.42	2.58	1.39	0.17	45.38	28.35	10.37	4.79
Indole	0.02	0.99	0.48	0.13	0.03	6.37	2.38	1.35	0.29
Diallyl trisulfide	–	1.18	0.33	0.28	–	3.42	0.89	0.45	0.17
Diallyl disulfide	–	0.58	0.22	–	–	13.09	6.89	3.53	1.07
Dimethyl trisulfide	–	–	0.06	–	–	1.02	0.17	–	–
Dimethyl disulfide	–	0.08	–	–	–	0.18	–	–	–
Methyl thiolacetate	–	0.29	0.14	–	–	1.13	0.28	0.09	–

Some compounds such as nonanal, octanol, 1‐hexanol, and 1‐octen‐3‐ol increased first and then decreased. There were also many compounds, such as (E,E)‐2,4‐heptadienal, 3‐methylbutyraldehyde, 2‐methylbutyraldehyde, 3‐methylbutyraldehyde, phenethyl alcohol, 2‐heptanone, trimethyl amine, pyrazine, ethyl acetate, and sulfur‐containing compounds that appeared in the middle or later stages of storage. For aldehydes, the peak areas of 3‐methylbutyraldehyde, 2‐methylbutyraldehyde, and hexanal with higher relative content increased gradually in the four groups; however, the relative content in CH + ε‐PL was 92.6%, 93.4%, and 93.3% lower than those in the control on day 12, respectively. (E,E)‐2,4‐heptadienal, which was not detected in the CH + ε‐PL samples, also increased in other groups during storage, while the peak areas were lower in the ε‐PL and CH group than in control at the end of storage. Similar results were observed in several other compounds, including phenethyl alcohol, 2‐ethyl‐1‐hexanol, 2‐heptanone, 2‐nonanone, trimethyl amine, pyrazine, indole, diallyl trisulfide, diallyl disulfide, and methyl thiolacetate. Some of these compounds were responsible for the off‐odor of the rotten shrimp (Zhou, Chong, Ding, Gu, & Liu, 2016). According to Parlapani, Mallouchos, Haroutounian, & Boziaris, 2014, the relative content in some compounds increased, and the production of new compounds was mainly because of the metabolism of spoilage microorganisms and the oxidation of fatty acids. The lower peak areas of 3‐methylbutyraldehyde, 2‐methylbutyraldehyde, hexanal, phenethyl alcohol, 2‐ethyl‐1‐hexanol, 2‐heptanone, 2‐nonanone, trimethyl amine, pyrazine, indole, diallyl trisulfide, and diallyl disulfide in treatments, especially in CH + ε‐PL samples, indicated that the metabolism of spoilage microorganisms and oxidation were effectively inhibited, and the flavor was effectively maintained by the coating. Similar results were reported by Yu, Regenstein, et al. (2018) for grass carp fillets with chitosan‐based coatings.

### 3.5 Texture properties

**Figure 5:**
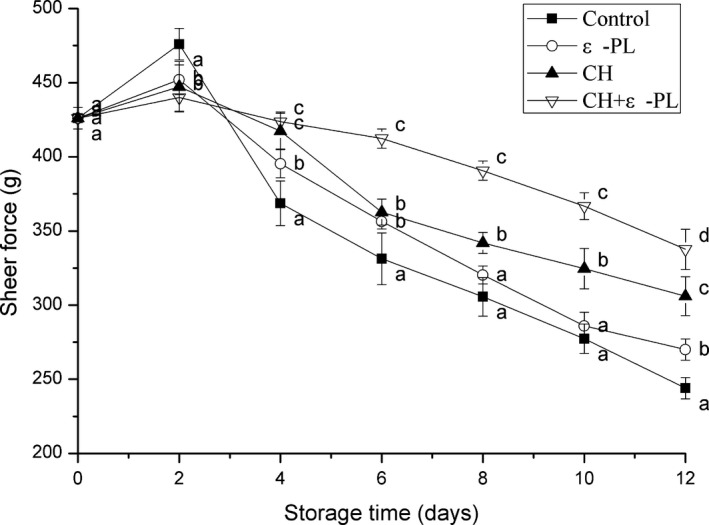
Changes in shear force of Chinese shrimp during refrigerated storage. Bars indicate means ± SD of 10 replicates. Different letters for the data points at each sampling point indicate significant differences (*p* < .05)

The texture of Chinese shrimp depends on the degradation of myofibrillar proteins during the storage, which is caused by the microbial decomposition and autolysis after the death of the shrimp. Shear force can be used as a reliable measure of the changes in the shrimp muscle tissue. As shown in Figure 5, shear force in the four groups slightly increased in the first two days, followed by a gradual decrease. The increase in the shear force during the early stages was caused by stiffness of the tissues after shrimp death, while the decrease was caused by autolysis and tissue degradation. Samples in the control group showed the fastest rate of decrease in shear force, dropping to 57.3%. While the rate of decline slowed down significantly in the coating groups, especially in the CH + ε‐PL group, indicating that the coating can delay the degradation of myofibrillar proteins caused by intrinsic biological factors, such as enzymes and microbes. Apparently, the texture of Chinese shrimp was improved by the coating treatment, while the CH + ε‐PL showed a better protective effect than CH or ε‐PL alone. Similar results showing that the texture deterioration of grass carp fillets was retarded by the chitosan‐based coating have been reported by Yu, Regenstein, et al. (2018).

## 4 CONCLUSIONS

The current findings indicated that the three treatments with 2.0% CH, 1.0% ε‐PL, or 2.0% CH and 1.0% ε‐PL could maintain the characteristic flavor and texture of refrigerated Chinese shrimp samples compared with the control treatment. Further, compared with treatment with CH or ε‐PL alone, treatment with CH + ε‐PL was optimal in maintaining acceptable characteristics of sensory and texture, reducing the relative content of off‐flavor compounds, including hypoxanthine, trimethyl amine, indole, and diallyl trisulfide. Therefore, chitosan combined with ε‐polylysine exhibits great potential as a preservation coating to maintain the flavor and texture quality of Chinese shrimp.

## CONFLICT OF INTEREST

The authors declare that they do not have any conflict of interest.

## ETHICAL APPROVAL

Ethical Review: This study does not involve any human or animal testing; Informed Consent: Written informed consent was obtained from all study participants.
